# Effects of Teriparatide Treatment on Bone Mineral Density in Patients With Osteoporosis: A Short-Term Dose-Response Study

**DOI:** 10.7759/cureus.45662

**Published:** 2023-09-21

**Authors:** Amarendra Nath Roy, Ipsita Mazumdar

**Affiliations:** 1 Department of Orthopaedics, Murshidabad Medical College, Berhampore, IND; 2 Department of Biochemistry, Jagannath Gupta Institute of Medical Sciences and Hospital, Kolkata, IND

**Keywords:** osteoporosis, bone mineral density, dexa score, recombinant parathyroid hormone, teriparatide

## Abstract

Introduction: Osteoporosis is a chronic bone disease that develops with a decrease in bone mineral density (BMD) along with bone mass. An essential measure in the prevention of osteoporosis is the achievement of normal peak bone mass. Teriparatide (TPTD) functions as an osteoanabolic agent, exerting a dual influence on bone remodeling by modulating both osteogenesis and osteoclastogenesis. Bone mass is determined by dual-energy x-ray absorptiometry (DEXA) quantitative CT scan and has outstanding precision (within 1% to 2%) and has the ability to show the effectiveness of drug intervention.

Objective: To assess the effectiveness of TPTD treatment on BMD in osteoporosis patients.

Materials and methods: The study is a retrospective, observational, multi-center study of 378 osteoporosis patients who were treated with 20 µg of subcutaneous TPTD every day for six months. DEXA scores of the hip joints of patients were measured both before and after TPTD treatment.

Results: The DEXA scores of the total population pre and post-treatment were -2.75+0.58 and -2.15+0.95 respectively, with a *p-value* of <0.001, which is statistically significant. In women the pre and post-treatment scores were -2.728+0.52 and -2.276+0.49, with a significant *p-value* of <0.001 whereas in males, the scores were -2.756+0.72 and -2.108+1.29 respectively, with a significant* p-value* of <0.05.

Conclusion: The six-month treatment with TPTD significantly improved DEXA scores in osteoporosis patients. DEXA score’s precision and sensitivity in quantifying impact contribute to effective osteoporosis management, guiding treatment strategies for better outcomes in the Indian population. Further research is warranted to see the long-term effect of TPTD.

## Introduction

Osteoporosis is a disorder characterized by increased bone turnover and decreased bone mass with associated skeletal fragility, resulting in an increased risk of fracture [1]. It is a well-defined and growing public health problem. The National Osteoporosis Foundation estimates that 10.2 million Americans have osteoporosis and an additional 43.4 million have low bone mass. It is estimated that by 2030, the number of adults with osteoporosis and low bone mass will increase to 71 million [1]. It is a frequent disease in postmenopausal women, which affects nearly one out of three women after the age of 50 years. Due to the aging of the population, the number of fractures is expected to increase by 25% in the next 10 years [2]. According to the statistics given by the World Health Organization (WHO), 30% of total post-menopausal women suffer from osteoporosis. There are more than 1.5 million fractures each year that are attributed to osteoporosis in America at a cost of US$14 billion to the US healthcare system [3]. Approximately 50% of women and 25% of men aged 50 years and above are at risk of experiencing a fracture linked to osteoporosis. The absolute risk of fracture rises twofold with each passing decade of life [4]. Although hip and vertebral fractures are commonly mentioned, a considerable portion of fractures consists of non-hip and non-vertebral fractures, which also carry significant morbidity [5].

Osteoporosis is typically diagnosed by assessing bone mineral density (BMD) using a noninvasive technique called dual-energy x-ray absorptiometry (DEXA) [6]. The current therapies for osteoporosis, including bisphosphonates, RANKL (receptor activator of nuclear factor kappa beta ligand) inhibitors, estrogen agonists/antagonists, parathyroid hormone (PTH) analogs, calcitonin, cathepsin K inhibitors, and sclerostin monoclonal antibodies, have certain limitations [7]. These can include rare side effects (such as osteonecrosis of the jaw, secondary hyperparathyroidism, and atrial fibrillation), the need for regular injections or administration, modest efficacy in reducing fracture risk, or restricted duration of use [8-10]. Teriparatide (TPTD), a PTH analog, stands out due to its ability to stimulate bone formation and significantly reduce both vertebral and non-vertebral fracture risk. It offers the convenience of once-daily subcutaneous injections and has shown efficacy in patients at high risk of fractures [11]. It manifests its favorable impacts on bone formation through dual modalities. The initial mode involves the direct instigation of bone formation within sites undergoing active remodeling (remodeling-associated bone formation), as well as on the surfaces of hitherto quiescent bone regions (modeling-associated bone formation). The secondary mode entails an elevation in the inception of novel remodeling sites. Both mechanisms collectively contribute to the eventual augmentation in bone density.

As per the current guidelines of the American College of Physicians on the treatment of osteoporosis, TPTD should be administered for a single course of 24 months and followed with an antiresorptive agent to maintain the gain in BMD [12]. Therefore, the present study aimed to assess the effect of TPTD treatment for six months, on BMD in osteoporosis patients by measuring DEXA scores before and after treatment.

## Materials and methods

This is a retrospective, observational, multi-center study of 378 patients from Murshidabad Medical College, Berhampore and Jagannath Gupta Institute of Medical Sciences and Hospital, Kolkata for a time duration of 52 weeks. Patients with various degrees of osteoporosis who were above 18 years old were included in the study. Patients who were active smokers, pre-menopausal women with a history of hysterectomy, or patients with a history of malignancy were excluded from the study. The included patients were treated with 20 µg of subcutaneous TPTD every day for six months. DEXA scores of the hip joints of patients were measured both before and after TPTD treatment.

DEXA scan was done to do BMD analysis involving T-score. A T-score shows how much the BMD of an individual varies from the BMD of an average young adult of the same sex, who has reached peak bone mass. Cut-off points were used for BMD T-scores indicating osteoporosis or low BMD. In the present study, T-scores of hip joints were taken in accordance with the World Health Organization (WHO) criteria. T-scores above -1 correspond to normal bone density, while T-scores falling between -1 and -2.5 indicate osteopenia, a condition featuring lower-than-normal bone density and a potential precursor to osteoporosis. T-scores below -2.5 signify the presence of osteoporosis, characterized by notably reduced bone density and heightened vulnerability to fractures [13].

Ethical clearance

An ethical waiver was granted by the institutional ethical committee due to the retrospective nature of the study.

Statistical analysis

The statistical analysis of the data was done using SPSS version 23 (IBM Corp., Armonk, NY). The primary endpoint was reported with two-sided 95% CIs, calculated using a paired t-test. A p-value of <0.05 is considered statistically significant.

## Results

A total of 378 individuals were included. Out of these, 72 (19%) were male and 306 (80.95%) were female. Other demographic details have been listed in Table 1.

**Table 1:** The demographic details of the study population

Variables	Value
Total, n (%)	378 (100%)
Male, n (%)	72 (19%)
Female, n (%)	306 (80.95%)
Age, years (mean ± SD)	63.26 ± 8.38
Indication	
Osteopenia patients, n (%)	130 (34.39%)
Osteoporosis patient, n (%)	248 (65.60%)

After six months of treatment with TPTD, female patients of both pre and post-menopausal age showed statistically significant improvement in DEXA scores (p < 0.001). However, there was no significant difference observed in DEXA scores for males. Also, there was no statistically significant association identified between the age of the patient and the difference in DEXA scores at six months of the treatment (p = 0.8993). The DEXA score analysis of the population has been listed in Table 2.

**Table 2:** DEXA scores of the hip joint of the population under study DEXA: dual-energy x-ray absorptiometry.

Subject	Mean + SD (pre-treatment)	Mean + SD (post-treatment)	P-value
Total (N = 378)	-2.8 ± 0.9	-2.3 ± 1.4	<0.001
Females (n = 306)	-2.8 ± 0.9	-2.3 ± 1.0	<0.001
Males (n = 72)	-2.7 ± 0.6	-2.5 ± 2.2	0.564

## Discussion

TPTD (recombinant human PTH (1-34)) and PTH (1-84) have now become pivotal components in osteoporosis treatment. PTH possesses the unique ability to activate both bone-resorbing (catabolic) and bone-building (anabolic) processes. The most prominent example of PTH's catabolic effects is observed in cases of primary hyperthyroidism. Predominant cortical skeletal involvement characterizes bone resorption in primary hyperparathyroidism, with a notable preservation of cancellous bone [14]. Remarkably, the judicious administration of low doses of TPTD or PTH (1-84) intermittently underscores that even under the circumstances of prolonged excessive PTH exposure inherent to this condition, the microarchitecture of cancellous bone in primary hyperparathyroidism remains upheld, if not augmented [15]. The anabolic influence of PTH on bone is multifaceted, encompassing pathways such as Wnt signaling (mediated through Wnt 10b stimulation), sclerostin inhibition, modulation of Runx2, and insulin-like growth factor (IGF-I), among others [16]. The net effect of intermittent low-dose PTH exposure initiates the recruitment of osteoblast progenitors and direct stimulation of mature osteoblasts. Furthermore, intermittent TPTD administration induces heightened bone turnover by stimulating osteoblast activity, leading to a pronounced elevation in the bone formation marker P1NP and accrual of bone density [17]. While TPTD also prompts bone resorption, its impact on bone formation over the initial 12-month period surpasses that of bone resorption. This culminates in a net increase in bone formation, thus fostering augmented bone mass and strength. Evidenced in clinical trials involving post-menopausal women, TPTD has demonstrated efficacy in enhancing BMD and mitigating clinical fractures, as contrasted with placebo [18] and oral bisphosphonates [19,20].

Solely, TPTD garnered approval from the US Food and Drug Administration (FDA) for the therapeutic intervention of osteoporosis in males. Additionally, it holds indications for managing osteoporosis induced by glucocorticoids [21]. Within its operational framework, TPTD elicits constructive influences on bone formation via two distinctive mechanisms. The initial mechanism encompasses the direct stimulation of bone formation, manifesting within locales of active remodeling (remodeling-centric bone formation), as well as along the surfaces of previously quiescent bone structures (modeling-centric bone formation). The secondary mechanism involves the heightened inception of fresh remodeling sites. The combined action of both processes substantiates the ultimate augmentation in bone density, as discerned through non-invasive assessment tools such as DEXA [5].

Numerous extensive observational investigations have been conducted on osteoporosis patients subjected to TPTD at a dosage of 20 μg/day. In these studies, TPTD is administered as a part of standard clinical care, rendering patient cohorts more reflective of real-world scenarios compared to the controlled parameters of randomized controlled trials. Notably, these observational TPTD studies lack control groups, thereby preventing direct comparison of fracture occurrences between TPTD-treated and other treatment cohorts. TPTD treatment is substantiated to yield advantageous outcomes, particularly in terms of enhancing BMD in critical areas such as the hips, spine, and femoral neck. As such, it stands as a favored intervention for mitigating the risk of complex fractures linked to these regions. The present study focuses on lumbar spine DEXA scores, chosen for their cost-effectiveness as an assessment metric. A 24-month investigation conducted by Miyauchi et al. in 2010 examined TPTD treatment effects among Asian participants. Daily administrations of 20 μg TPTD were administered to 96 Japanese individuals, both men and women, characterized by low bone mass. The findings unveiled noteworthy enhancements from baseline in areal BMD, showcasing increases of +13.42% at the spine, +3.67% at the total hip, and +3.26% at the femoral neck [22]. The utilization of areal BMD assessments via DEXA at the spine and proximal femur, standard techniques for diagnosing osteoporosis, remains integral. Furthermore, the response to therapeutic interventions is often gauged through sequential BMD evaluations [23,24].

A meta-analysis compared the efficacy of TPTD and bisphosphonates for reducing vertebral fracture risk and BMD in the lumbar spine and femoral neck in postmenopausal women with osteoporosis. The results indicated that TPTD was associated with a reduction of the vertebral fracture risk (risk ratio (RR) = 0.57, 95% confidence interval (CI): 0.35, 0.93, p = 0.024). Furthermore, TPTD therapy increased the mean percent change in BMD in the lumbar spine at six months, 12 months, and 18 months as compared to bisphosphonates with p< 0.05 [25]. Shin et al. (2019) demonstrated that TPTD led to a reduction in the duration of bone union. The TPTD group exhibited an average bone union time of 18 weeks, notably shorter than the 23-week average observed in the non-TPTD group (p* *= 0.001) [26]. A retrospective review was conducted on the medical records of 34 Japanese patients who received oral bisphosphonates and experienced a total of 45 atypical femoral fractures (AFFs). The study found that the TPTD group had a significantly shorter average fracture union time (5.4 ± 1.5 months) compared to the non-TPTD group (8.6 ± 4.7 months; p = 0.012). Additionally, the TPTD group had a significantly lower frequency of delayed healing or non-union compared to the non-TPTD group (p = 0.014) [27]. A separate retrospective study indicated the potential of TPTD treatment to support fracture healing in patients with AFFs. Within the TPTD group (comprising eight cases), the mean duration for achieving bone union was 4.4 months, which contrasted with the non-TPTD group (also eight cases) exhibiting an average of 6.2 months (p = 0.116) [28]. The effect of TPTD to increase bone formation, as demonstrated by studies of iliac crest biopsies, bone scans, and positron emission tomography, would be anticipated to increase bone mass and hence BMD.

Furthermore, there are various real-world studies that state the effectiveness of TPTD in treating various patient populations in the last two decades. In post-menopausal women, TPTD has demonstrated effectiveness in improving bone density and reducing fractures, especially vertebral ones, compared to placebo and oral bisphosphonates. However, its superiority for non-vertebral fractures remains uncertain. In men, TPTD shows promising results with significant gains in bone density. In glucocorticoid-induced osteoporosis, TPTD is considered when patients experience fractures despite antiresorptive treatment and early use may be explored for long-term high-dose glucocorticoid users. Despite concerns about patient response variability, safety, and potential osteosarcoma risks, real-world data reaffirm the benefits of TPTD in reducing fracture risk across different patient populations, emphasizing its role as a valuable option in osteoporosis management [11].

The present study is the first of its kind in the Indian population that quantitatively measures the effects of TPTD on BMD through DEXA scores in the Indian population, and it shows corroborative results with all previous studies throughout the world. It offers valuable insights into the subject matter and contributes significantly to the existing body of knowledge, despite certain limitations that should be acknowledged. These limitations primarily include the retrospective design and the absence of a control group. While retrospective studies may be influenced by inherent biases and confounding variables, the findings from our study provide valuable information for understanding the topic at hand. It is important to note that the use of medical records for data collection, despite the possibility of incomplete or missing data, still allowed us to gather substantial information for analysis. Overall, our study adds to the scientific understanding of the field and highlights areas for further investigation.

## Conclusions

In brief, the study revealed that after six months of TPTD treatment, there was a statistically significant improvement in DEXA scores observed in osteoporosis patients indicating improved bone health. The precision and sensitivity of DEXA scores in quantifying impact in enhancing osteoporosis management, shaping improved treatment strategies for favorable outcomes in the Indian population. Subsequent research is needed to explore the long-term effect of TPTD treatment.
